# Vasculoprotective Effects of 3-Hydroxybenzaldehyde against VSMCs Proliferation and ECs Inflammation

**DOI:** 10.1371/journal.pone.0149394

**Published:** 2016-03-22

**Authors:** Byung Soo Kong, Soo Jung Im, Yang Jong Lee, Yoon Hee Cho, Yu Ri Do, Jung Woo Byun, Cheol Ryong Ku, Eun Jig Lee

**Affiliations:** 1 Brain Korea 21 PLUS Project for Medical Science, Yonsei University, Seoul, Korea; 2 Endocrinology, Institute of Endocrine Research, College of Medicine, Yonsei University, Seoul, Korea; Centro Cardiologico Monzino, ITALY

## Abstract

3-hydroxybenzaldehyde (3-HBA) is a precursor compound for phenolic compounds like Protocatechuic aldehyde (PCA). From recent reports, PCA has shown vasculoprotective potency, but the effects of 3-HBA remain unclear. The aim of this study is to investigate the vasculoprotective effects of 3-HBA in endothelial cells, vascular smooth muscle cells and various animal models. We tested effects of 3-HBA in both vitro and vivo. 3-HBA showed that it prevents PDGF-induced vascular smooth muscle cells (VSMCs) migration and proliferation from MTS, BrdU assays and inhibition of AKT phosphorylation. It arrested S and G0/G1 phase of VSMC cell cycle in PI staining and it also showed inhibited expression levels of Rb1 and CD1. In human umbilical vein endothelial cells (HUVECs), 3-HBA inhibited inflammatory markers and signaling molecules (VCAM-1, ICAM-1, p-NF-κB and p-p38). For ex vivo, 3-HBA has shown dramatic effects in suppressing the sprouting from aortic ring of Spargue Dawley (SD) rats. In vivo data supported the vasculoprotective effects of 3-HBA as it inhibited angiogenesis from Matrigel Plug assay in C57BL6 mouse, prevented ADP-induced thrombus generation, increased blood circulation after formation of thrombus, and attenuated neointima formation induced by common carotid artery balloon injury of SD rats. 3-HBA, a novel therapeutic agent, has shown vasculoprotective potency in both in vitro and in vivo.

## Introduction

Atherosclerosis is a multi-factor disease process including outgrowth and migration of vascular smooth muscle cells (VSMCs), endothelial dysfunction, chronic inflammation, plaque formation, and rupture and arterial thrombosis. Previous studies on atherosclerosis have shown that the disease is characterized by the increased production of proteins such as VCAM-1, ICAM-1, phosphorylated AKT, CD1, and matrix metalloproteinases (MMPs) and the generation of reactive oxygen species (ROS).[1–5] Also, increased VSMC proliferation and migration, increased expression of adhesion molecules on the surfaces of endothelial cells, augmented platelet aggregation, and inflammatory angiogenesis have been observed.[6–9]

Benzaldehydes, which have a myriad of applications in cosmetics, as flavoring agents, and as fragrances, are “generally regarded as safe” (GRAS) by the United States FDA.[10] The latest findings show that these compounds have therapeutic effects for the treatment of various diseases such as cancer and vascular and renal diseases.[11–14] With regard to atherosclerosis, benzaldehydes are reported as potent inhibitors of lipoprotein-associated phospholipase A2[15] and are capable upregulators of ABCA1[16].

*3*-Hydroxybenzaldehyde (*3*-HBA) is one of the benzaldehydes commonly found in nature. It is produced by 3-hydroxybenzyl-alcohol dehydrogenase,[17] and is a substrate of aldehyde dehydrogenase (ALDH) in rats and humans (ALDH2).[18, 19] It is widely used in the chemical synthesis of flavonoids, which are well-known antioxidants. It is a structural isomer of salicylaldehyde and *4*-HBA and has one hydroxyl (OH) group at the *meta* position of the phenolic ring. Several reports have mentioned the importance of OH group position in hydroxybenzaldehydes. Reports from Cao *et al*. suggest that benzaldehydes with OH groups have higher intracellular antioxidative activity than those without and showed that methylated or glycosylated C3-OH no longer exhibits antioxidative activity.[20, 21] *3*-HBA has been used as a precursor agent for the synthesis of PET agent (J147) in Alzheimer’s disease, but there are very few reports delineating the therapeutic effects of *3*-HBA in atherosclerosis.[12, 22]

Here, we show that treatment of *3*-HBA has vasoprotective effects in both vitro and vivo. The vasoprotective effects of *3*-HBA involve anti-proliferative and anti-migrative effects on VSMCs and ROS inhibition and p-p38/p-p65 inhibition of HUVECs. Also, we observed these effects in both *ex vivo* and *in vivo* levels by assessing *3*-HBA on anti-angiogenic and anti-thrombic effects. Finally, we showed that *3*-HBA has inhibitory effect on the formation of Neointima.

## Materials and Methods

### Reagents

*3*-Hydroxylbenzaldehyde (*3*-HBA) was purchased from Sigma Aldrich (St. Louis, MO, USA), dissolved in water, and filtered using a 0.2-μm pore cellulose acetate syringe filter (16534-K; Sartorious, Goettingen, Germany). TNF-α and rat PDGF-BB were purchased from R&D Systems (Minneapolis, MN, USA). Heparin and normal saline were purchased from JW Pharmaceutical (Seoul, Korea). Antibodies for Western blot analysis against VCAM-1 (sc-8304), ICAM-1 (sc-7891), MMP-2 (sc-10736), and CD31 (sc-1506) were purchased from Santa Cruz (Delaware, CA, USA). Phospho-AKT (#9271S), AKT (#9272S), phospho-MAPK (#9106S), NFκB (#4767), and phospho-NFκB (#3033S) were purchased from Cell Signaling Technology (Danvers, MA, USA). Cultrex BME Matrigel (3431-005-01) was purchased from Trevigen (Gaithersburg, MD, USA) for use in the sprout ring assay. MTS assay kit (G3580) was purchased from Promega (Madison, WI, USA). Propidium iodide (PI) was purchased from Sigma Aldrich for use in cell cycle assay. Bromodeoxyuridine (BrdU) incorporation assay kit was purchased from Roche (Basel, Switzerland) for use in cell proliferation ELISA assay.

### Cell culture

Primary human umbilical vein endothelial cells (HUVECs) were purchased from GIBCO (C-003-5C), cultured, and prepared for experiments as reported in a previous study. Rat vascular smooth muscle cells (VSMCs) were isolated from the thoracic aortas of Sprague Dawley (SD) rats (250–300 g; Orient Technology, Seoul, Korea) as it is previously described,[13] anesthetized with Zoletil 30 mg/kg (Virbac) and Rompun 10 mg/kg (Bayer) by intraperitoneal (i.p.) injection. After the procedure, rats were euthanized via carbon dioxide inhalation. The VSMCs in passages 3–6 were used in experiments after serum deprivation for 24 h. [23]

### Reverse transcription-polymerase chain reaction (RT-PCR)

Total RNA was extracted from VSMC lysates with Isol RNA lysis reagent (5 prime, Hilden, Germany), and cDNA was prepared using ReverTra Ace -α- ® (Toyobo, Osaka, Japan) as described previously.[24, 25] Primers designed for cyclin D1 were based on the rat CCND1 gene sequence (forward primer: 5’-CCTGACTGCCGAGAAGTTGT; reverse primer: 5’-TCATCCGCCTCTGGCATTTT), the rat RB1 gene (forward primer: 5’-AACTCTGGGGCATCTGCATC; reverse primer: 5’-TTGCAGCTGTTTTGTACGGC), human HO-1 gene (forward primer. 5’-TCCGATGGGTCCTTACACTC; reverse: 5’-ATTGCCTGGATGTGCTTTTC),[26] and human NRF gene (forward primer. 5’-CGGTATGCAACAGGACATTG; reverse: 5’-ACTGGTTGGGGTCTTCTGTG). Oligonucleotide primers were purchased from Bioneer (Seoul, Korea). PCR products were resolved in 2% agarose gel via electrophoresis.

### Western blotting

VSMCs and HUVECs were lysed and prepared for Western blot analysis using antibodies against MMP-2, phospho-AKT, AKT, VCAM-1, ICAM-1, phospho-MAPK, and phospho-NFκB as described earlier. After incubation with a specific secondary antibody coupled to horseradish peroxidase, blots were visualized using enhanced chemiluminescence (Ab-frontier, Seoul, Korea).

### *In vitro* assays

#### Propidium iodide staining for cell cycle analysis

Procedures were performed as described in an earlier report. VSMCs were stained with PI staining solution at room temperature for 30 min. Cell cycle analysis was evaluated using a flow cytometer (Becton Dickinson, FACS Calibur) and analyzed with the FlowJo program.

#### Cell migration assay

When VSMCs and HUVECs reached 80% confluence in 6-well plates, the single-cell layer was scratched with a sterile plastic 1000-μl tip. After, media was changed into serum free media for 24 h and then, cells were pretreated with *3*-HBA for 24 h. Cell migration was photographed using a Nikon microscope system (Nikon Instrument). The area of wound healing was measured using Image J software.

#### Cell viability and proliferation assay

MTS assay procedures were modified from previous reports.[26, 27] VSMCs and HUVECs were serum deprived, and then *3*-HBA in serum-free media was added to each group. After 24 h of *3*-HBA treatment, the media was replaced with PDGF added to *3*-HBA-containing media in all except the control group. After 24 h, MTS reagent concentration was measured as absorbance. BrdU incorporation analysis was performed for further investigation. All procedures were performed according to the manufacturer’s instructions. Absorbance was measured at 450 nm. Data were analyzed with t-test by SPSS program.

#### Measurement of reactive oxygen species in HUVECs and VSMCs

Reactive oxygen species (ROS) procedures were performed as described in a previous report. Levels of cellular reactive oxygen species were measured using the fluorescent probe 5-(and-6)-chloromethyl-2’, 7’-diflurodihydrofluorescein diacetate (CM-H2DFFDA). To prepare samples for ROS assay, HUVECs were cultured in EBM-2 supplemented with serum kit and then it was changed in to serum free media with 0.1% serum. *3*-HBA is treated for 24 hours and then, 1 hour of H_2_O_2_ 100 μM is added. Samples were analyzed via FACS Calibur flow cytometry.

### *Ex* and *in vivo* assays

#### Blood aggregation

The sample blood for *ex vivo* analysis was obtained from 6-week-old male SD rats (Orient-Charles River Technology, Gyunggido, Korea) and collected in 1.0% heparin to prevent coagulation.[28] Sample blood for *in vivo* testing was obtained from the animals treated with i.p. 100 mg/kg *3*-HBA (n = 6) and 100 mg/kg aspirin (n = 6) daily for 1 week. Platelet aggregation was induced by the addition of 20 μM ADP (Sigma–Aldrich Co., MO, USA) and was later evaluated with an impedance aggregometer (Chrono-log model 700, Chronolog Corporation, Havertown, PA, USA).

#### SD rat models for tail vein thrombosis

To evaluate the effect of *3*-HBA on thrombosis formation, tail thrombosis was induced in 6-week-old male SD rats that were treated with 100 mg/kg *3*-HBA for 1 week, as described earlier. Animals were injected intravenously with 1 mg/kg κ-carrageenan in order to induce tail vein thrombosis. After injection, the tail region 13 cm from the tip was ligated for 10 minutes and then freed. The heparin group (n = 6) was injected with 200 IU of heparin and used as the positive control.

#### Animal model–common carotid balloon injury

Male SD rats weighing 200~225 g were randomly divided into three groups as follows: Group 1 –Sham operated; Group 2 –Balloon injury; Group 3–*3*-HBA (100mg/kg) with balloon injury. For operative procedures, SD rats were anesthetized with 5% isoflurane in a mixture of 70% N_2_O and 30% O_2_ that was maintained in 2% isoflurane.[12] Animals were i.p. injected, with specific formulation and volumes as it was previously reported[12], for 2 weeks before the operative procedure and an additional 4 weeks after 2 days of recovery. In order to obtain the aortas for analysis, the animals were anesthetized using Zoletil (30 mg/kg) and Rompun (10 mg/kg) by i.p. injection and were sacrificed by carbon dioxide inhalation.

#### Sprout ring assay

Male Sprague Dawley Rats (100g) were housed in a controlled environment and after one week of stabilization, under anesthesia with Zoletil (30 mg/kg) and Rompun (10 mg/kg) by i.p. injection, rats were sacrificed and thoracic aortas were obtained. Thoracic aortas were chopped into several pieces and placed on Cell Culture Insert (PICM03050) purchased from Millicell (Billerica, MA, USA) in 6-well plate. EBM-2 serum media was added into the dish and incubated for 3 days. Then, *3*-HBA was added, and the media was replaced every 2 days with substances. At day 7, aortas were photographed using an Olympus microscope at an appropriate magnification.

#### In vivo Matrigel plug assay

The Matrigel plug assay was performed as previously described.[9] In brief, 20-g and 7-week-old C57BL/6 mice (Orient Technology, Seoul, Korea) were injected subcutaneously with 0.6 ml of Matrigel containing the indicated amount of *3*-HBA and 100 ng/ml of endothelial cell growth (ECG) supplement (354006, BD Bioscience, Bedford, MA, USA). After 6 days, the mice were anesthetized using Zoletil (30 mg/kg) and Rompun (10 mg/kg) by i.p. injection. Then, the skin of the mouse was pulled back to expose the Matrigel plug, which remained intact. The animals were then euthanized by carbon dioxide inhalation. Hemoglobin obtained from the Matrigel was measured using the Drabkin reagent kit 525 (Sigma-Aldrich) in order to qualify blood vessel formation. The concentration of hemoglobin was calculated in comparison to a known amount of hemoglobin assayed in parallel. To identify infiltrating endothelial cells (ECs), immunohistochemistry was performed using anti-CD-31 antibody (Santa Cruz).

### Vascular histology and immunohistochemical procedures

Rat aortas were prepared for immunohistochemical analyses in 4-μm paraffin cross-sections and were stained with hematoxylin and eosin using a standard protocol.[12, 29] Sections were blocked with 5% donkey serum (017-000-121, Jackson ImmunoResearch, West Grove, PA, USA) in antibody diluent (S2022, Dako, Glostrup, Denmark) for 30 min. Then, sections were incubated overnight at 4°C with the following antibodies: ICAM-1, CD31, and VCAM-1 (1:150). Slides were incubated for 1 h with a biotinylated secondary antibody (Vector Laboratories, Burlingame, CA, USA). After rinsing three times in TBS-T, RTU horseradish peroxidase streptavidin (SA5704, Vector Laboratories) was applied, and the slides were incubated for 10 min. For color development, 3,3’-diaminobenzidine (DAB, D5637, Sigma) was used.

### Statistical analysis for *in vitro* results

For in vitro results, PCR and Western blots were measured by using densitrometer (MiniBIS Pro). The images were then quantify ed by using Genetools (Syngene) software. After the blots were quantified, results and t-tests were evaluated and analyzed using SPSS 18.0 software.

### Stastical analysis for *ex vivo* and *in vivo* results

To perform image analysis for *ex vivo* and *in vivo* results, all images were collected under the same observation conditions (light, contrast, magnification). Differences between groups for both *in vivo* and *ex vivo* results were evaluated using SPSS 18.0 software. For morphologic analysis of neointimal formation, Scion Image software was used.,The results are expressed as mean ± SEM.six round cross-sections (4-μm thickness) were cut from the approximate middle of the artery. The intimal and medial cross-sectional areas of the carotid arteries were measured to calculate neointima size (inches squared). Graphs of the balloon injury and sprout ring assays were produced using the MedCalc software program.

### Ethics statement

All animal procedures were reviewed and approved by the Institutional Animal Care and Use Committee (IACUC) of Yonsei University Health System (approval number: 2010–0268) and were performed in strict accordance with the Association for Assessment and Accreditation of Laboratory Animal Care and the NIH guidelines (Guide for the Care and Use of Laboratory Animals).

## Results

### *3*-HBA inhibits VSMC proliferation and cell cycle

**Fig 1:**
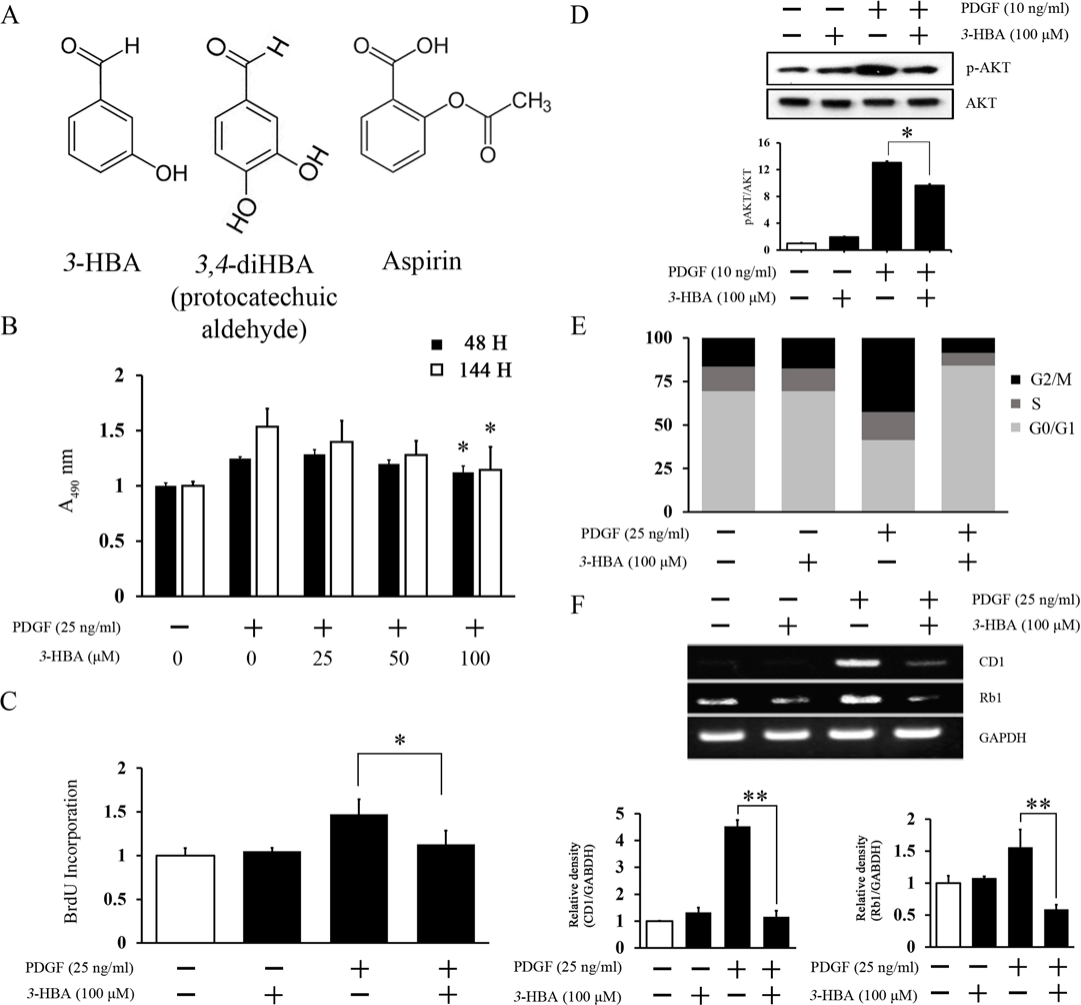
(**A**) Chemical structures of *3*-HBA, PCA, and aspirin. (**B**, **C**, **D**) The inhibitory effect of *3*-HBA on PDGF-induced proliferation in rat VSMCs. VSMCs were prepared for experiments before 24 h serum starvation. (**B**) For MTS assay, VSMCs were pretreated with *3*-HBA (0, 25, 50, 100 μM) in a concentration-dependent manner for 24 h and then stimulated with PDGF (25 ng/ml) for 48 and 144 h. (**C**) For BrdU assay, VSMCs were pretreated with *3*-HBA (0, 100 μM) for 24 h and then stimulated with PDGF (25 ng/ml) for 48 h. (**B**, **C**) Cell proliferation was measured at 490 nm. (**D**) Phosphorylation of AKT was determined by Western blot analysis. (**E**, **F**) The inhibitory effect of *3*-HBA on the cell cycle of rat VSMCs. VSMCs were serum starved for 24 h and then pretreated with *3*-HBA (0, 100 μM) for 24 h. VSMCs were then stimulated with PDGF (25 ng/ml) for 24 h. (**E**) Cell cycle distribution was measured with PI staining. (**F**) Gene expression was analyzed by RT-PCR. Each result is expressed as the mean ± standard error. * indicates *p* < 0.05 compared to the PDGF group. ** indicates *p* < 0.005 compared to the PDGF group. Values represent the mean ± SEM of three independent sets of experiments.

To confirm the anti-proliferative and vasoprotective effect of *3*-HBA (Fig 1A) on PDGF-induced VSMC proliferation, BrdU assay was performed. Firstly, we checked with MTS assay whether concentration of *3*-HBA (0, 25, 50, 100 μM) is not toxic to the cell at different incubation time and concentration (Fig 1B). Treatment of *3*-HBA showed low toxicity in VSMC for both 48 and 144 h. Also, recent reports show that NSAID (nonsteroidal anti-inflammatory drugs) taken in large dose could cause some severe damage to individuals by affecting on the cell cycle.[30] Comparison between 3-HBA and control groups showed similar cell cycle condition indicating that the treatment of 3-HBA did not alter VSMC cell cycle (Fig 1E).

Inhibition of VSMC proliferation is essential to treat atherosclerosis that BrDu assay and PI staining were performed. *3*-HBA has decreased the BrdU incorporation in comparison to PDGF (Fig 1C). Consistent with these data, *3*-HBA inhibited AKT phosphorylation (Fig 1D). Based on these results, *3*-HBA shows suppressive effects on PDGF-induced VSMC proliferation. PI staining show that *3*-HBA could arrest the S phase and G0/G1 phase, which were increased by PDGF (Fig 1E). Furthermore, the expression levels of cyclin D1 (CD1) and retinoblastoma (Rb1) mRNA, the representative markers of cell cycle regulation,[31] were increased by PDGF stimulation; however, *3*-HBA treatment lowered these expression levels in VSMCs (Fig 1F).

### *3*-HBA inhibits VSMC cell migration

**Fig 2:**
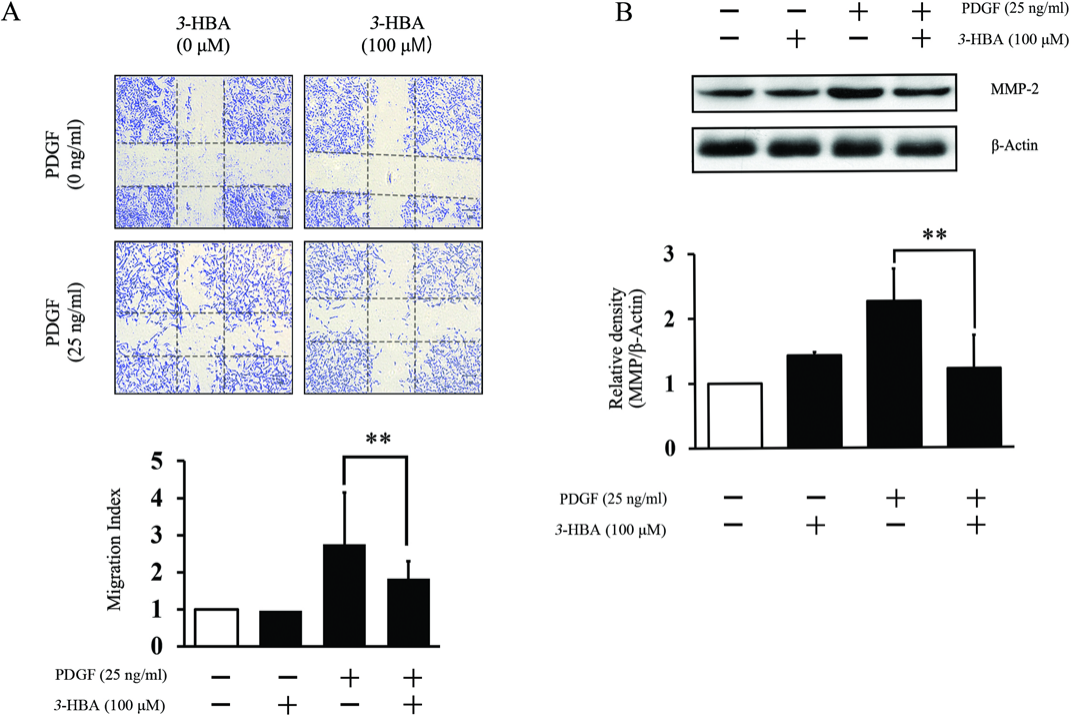
The suppressive effect of *3*-HBA on rat VSMC migration. (**A**, **B**) VSMCs were serum starved for 24 h and then pretreated with *3*-HBA (0, 100 μM) for 24 h. VSMCs were stimulated with PDGF (**A,B**) 25 ng/ml for 24 h. (**A**) Before stimulation, wells were scratched, and cells were stained with hematoxylin and eosin and scored using Image J. (**B**) MMP-2 protein levels were determined by Western blot analysis. ** indicates *p* < 0.005 compared to the PDGF group. Values represent the mean ± SEM of three independent sets of experiments.

Assessment of the migration of VSMCs is an important criterion to evaluate vasculoprotective effect of *3*-HBA, we performed wound-healing experiments. The results in representative images show that *3*-HBA inhibited the migration of VSMCs (Fig 2A). In accordance with the image, the graph shows that *3*-HBA decreased the number of migrated cells. Moreover, the production of MMP-2, an important protein marker of the migration of cells,[4] was also significantly decreased in the *3*-HBA-treated group (Fig 2B). However, the treatment of *3*-HBA in HUVECs did not show any effects in migration of endothelial cells (S1 Fig)

### *3*-HBA inhibits inflammatory signaling in HUVECs

**Fig 3:**
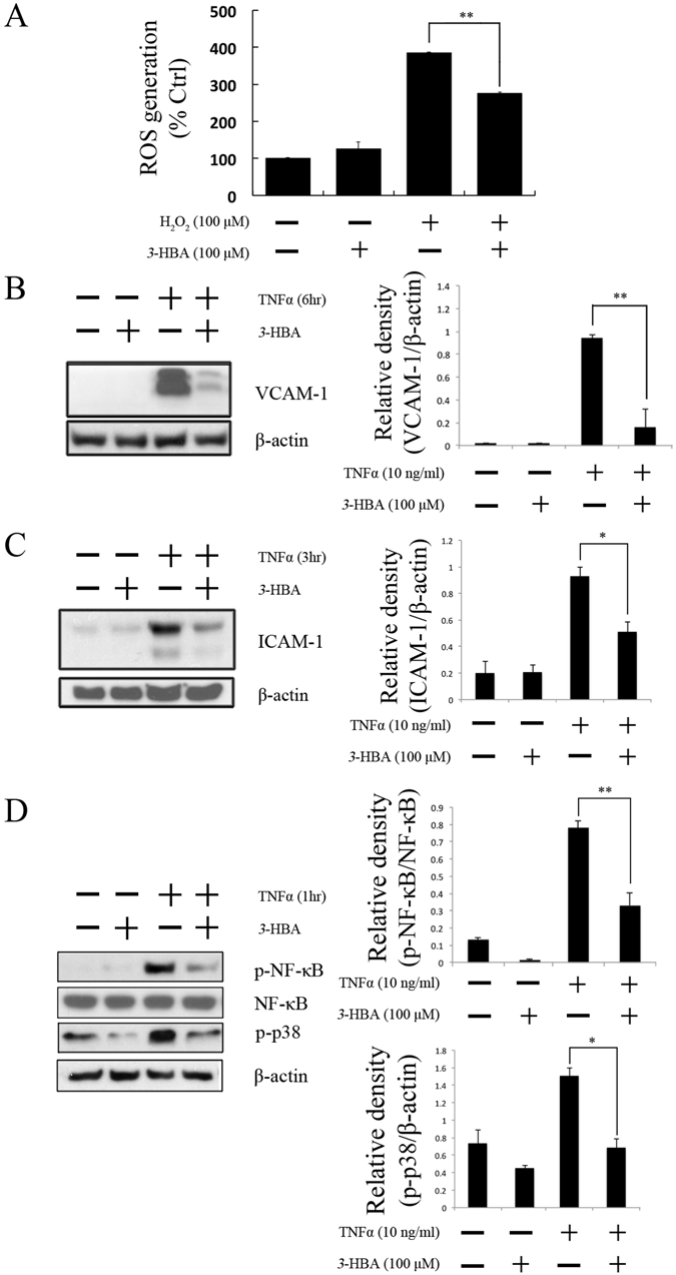
*3*-HBA inhibits inflammation induced by TNF-α in HUVECs. (**A, B, C, D**) HUVECs were pretreated with *3*-HBA (100 μM) for 24 h. Treatment with H_2_O_2_ (100 μM) for 1 h (**A**) and with TNF-α (10 ng/ml) for (**B**) 6 h, (**C**) 3 h, and **(D)** 1 h. (**B, C**) Densitometric analyses are presented as the relative ratio of VCAM-1, ICAM-1, or phospho-p38 to β-actin. (**D**) Densitometric analyses of phospho-NF-κB are presented as the relative ratio to NF-κB. Values represent the mean ± SEM of three independent sets of experiments; ** indicates *p* < 0.005.

To assess whether *3*-HBA has vasoprotective and anti-inflammatory effects in HUVECs, we first evaluated the effect of *3*-HBA on ROS production. HUVECs were pretreated with *3*-HBA for 24 h, followed by H_2_O_2_ (100 μM) treatment for 1 h. Results show that *3*-HBA has ROS inhibitory effects (Fig 3A). Also the expression of NRF-2 and HO-1, which are closely related with ROS inhibition, have been increased by the treatment of *3*-HBA in HUVECs (S2 Fig). However, no effects were observed from VSMCs on HO-1 and NRF2 (data not shown). These data show that *3*-HBA exhibits its anti-inflammtory effects uniquely through endothelial cells. As it is well known that ROS is one of the factors causing inflammation, we further assessed the effect of *3*-HBA on inflammatory protein production. HUVECs were pretreated for 24 h with *3*-HBA, followed by treatment with TNF-α (10 ng/ml) for 1, 3, and 6 h depending on the different targets (VCAM-1, ICAM-1, p-NF-κB, or p-p38). TNF-α stimulated ICAM-1 and VCAM-1 within 3 h and in 6 h, respectively. However, *3*-HBA pretreatment significantly downregulated these production levels in HUVECs (Fig 3B and 3C). To investigate the signaling pathway used by *3*-HBA to downregulate these inflammatory proteins, we analyzed the production of p-NF-κB and p-p38. HUVECs were pretreated under the same conditions as before and then treated with TNFα (10ng/ml, 1h). Data show that the increased production levels of p-NF-κB and p-p38 induced by TNFα were inhibited by pretreatment with *3*-HBA in HUVECs (Fig 3D).

### *3*-HBA inhibits *ex vivo* and *in vivo* angiogenesis

**Fig 4:**
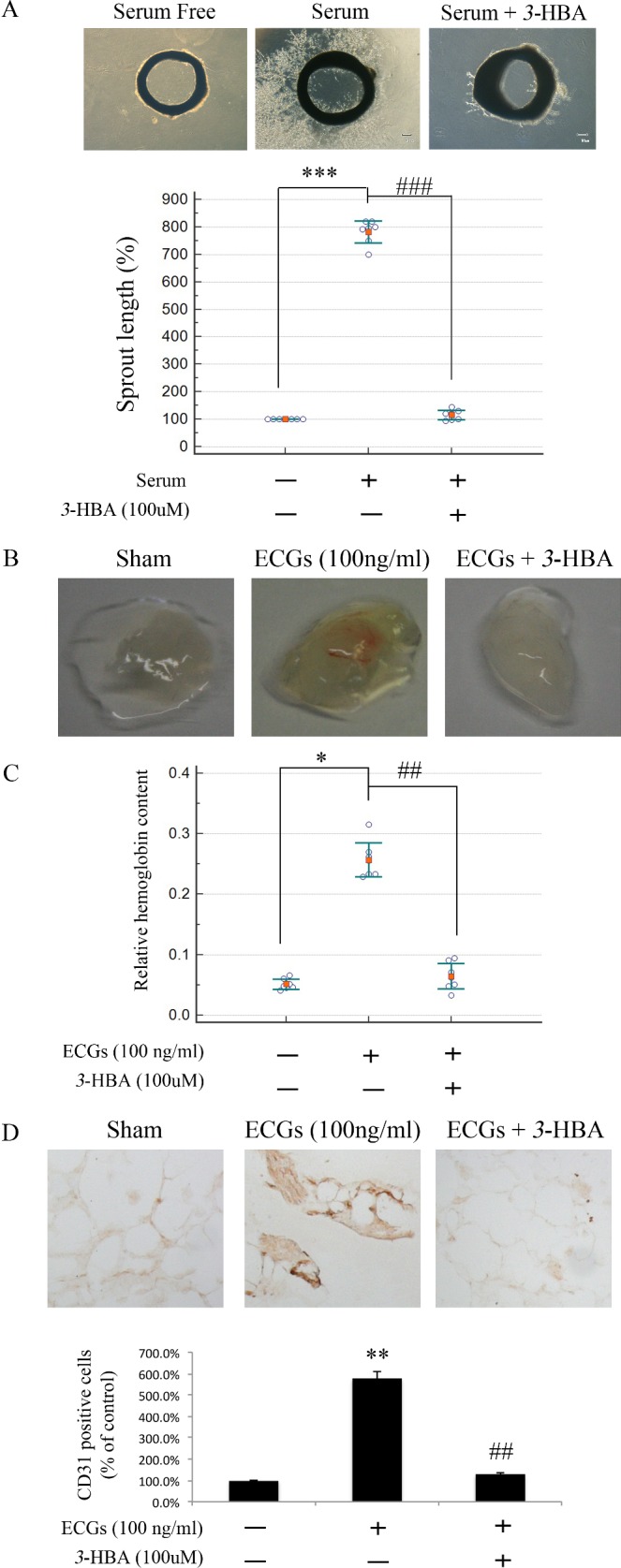
*3*-HBA inhibits angiogenesis *ex vivo* and *in vivo*. (**A**) Representative images of sprout ring assays are shown. Aortic segments used for each set of experiments were derived from single SD rats weighing 100 g. For analysis and quantification, three aortic segments were used for each group (*n* = 3). Harvested aortic segments in Matrigel were stimulated with FBS (10%) for 3 days. Then, the segments were treated with *3*-HBA (100 μM) for 48 h in media supplemented with FBS (10%). Quantification of sprouting was measured using Scion Image software. Values represent the mean ± SEM of three experiments. *** indicates *p* < 0.005 compared to the serum-free group. ### indicates *p* < 0.005 compared to the serum group. (**B**) Representative Matrigel plugs were photographed (*n* = 3 in each group). (**C**) Quantification of hemoglobin content was conducted in Matrigel plugs that were stained for infiltrating ECs with anti-CD31 antibody. * indicates *p* < 0.01 compared to the sham group. ## indicates *p* < 0.05 compared to the ECG group. (**D**) The graph shows quantitative assessment of CD31^+^ ECs. Values represent the mean ± SEM of three experiments.

Previous results have shown that *3*-HBA has vasoprotective effects *in vitro*. We next investigated the anti-angiogenic activity of *3*-HBA in both *ex vivo* and *in vivo* angiogenesis models. *Ex vivo* model of Sprout ring assay shows that serum condition could increase the growth of aortic sprouts. However, treatment with *3*-HBA significantly prevented the growth of aortic sprouts compared to the serum control group. Consistent with this observation, sprout length was significantly decreased (Fig 4A).

To confirm the anti-angiogenic effect of *3*-HBA *in vivo*, we performed Matrigel plug assays. Matrigel plugs containing ECGs were red in color as the result of neovascularization, but the Matrigel plugs treated with *3*-HBA were white in color, like the sham-operated control (Fig 4B). To confirm this observation, hemoglobin was quantified in each Matrigel plug. The results show that the Matrigel plugs containing ECGs had more hemoglobin than the sham-operated control. Furthermore, treatment with *3*-HBA in ECG-containing Matrigel plugs decreased the hemoglobin content (Fig 4C). The Matrigel plugs were immunohistochemically stained with anti-CD31 for vessel density analysis. The results showed a lower functional vasculature density in the *3*-HBA-treated plugs than the ECG-treated control plugs (Fig 4D). Interestingly, 4-HBA, a stereoisomer of *3*-HBA, showed higher intensity of redness than the ECG containing Matrigel (S3 Fig). This suggest the position of–OH in hydroxylbenzaldehyde might play a key role in the exhibition of vasculoprotective effects.

### *3*-HBA exhibits anti-thrombic effects

**Fig 5:**
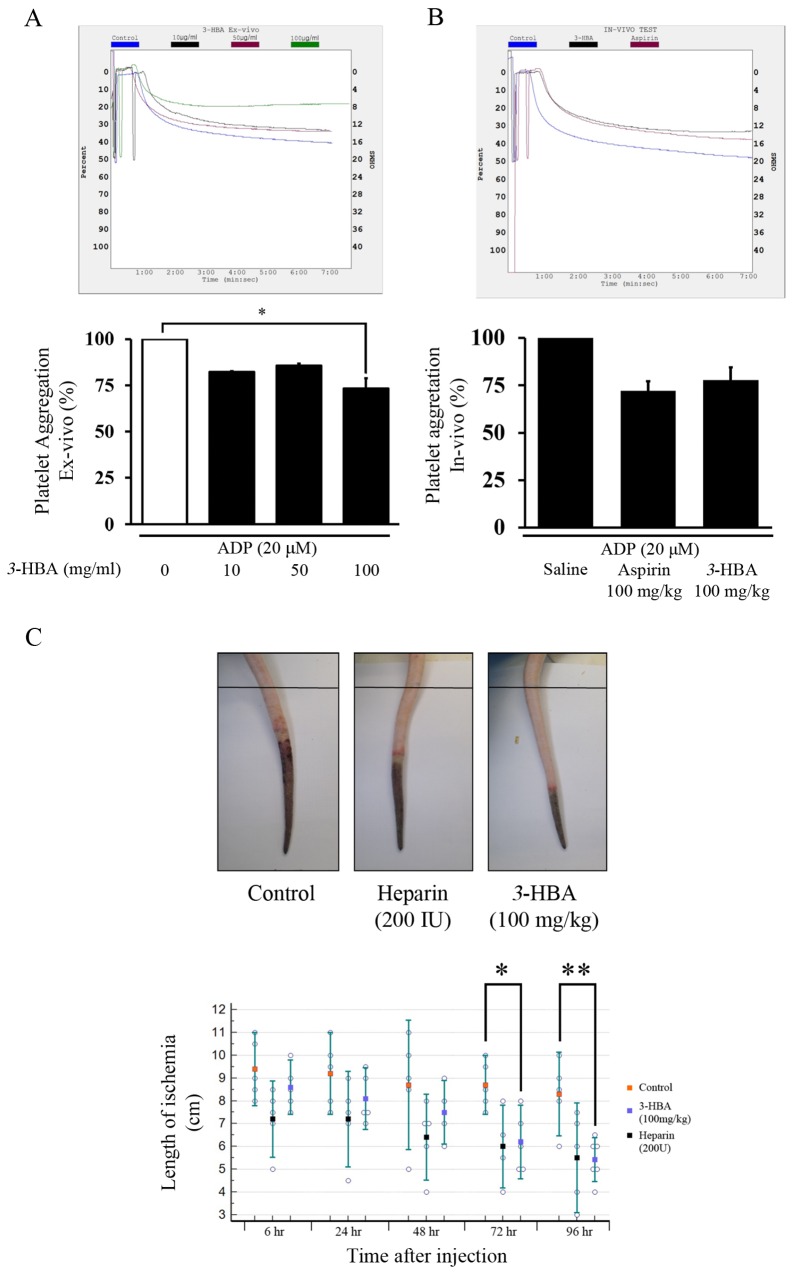
The blood thinning and restoration effect of *3*-HBA on SD rats after aggregation. (**A**, **B**) The sample blood was collected from 7-week-old male SD rats. *3*-HBA and aspirin were administered at a dose of 100 mg/kg/ for 1 week by i.p. injection (*n* = 6 in each group). ADP was induced for platelet aggregation after *3*-HBA treatment in (**A**) *ex vivo* and (**B**) *in vivo* conditions. The top graph shows the impedance aggregometer mean value of each group, and the bottom bar graph represents aggregation velocity. (**C**) *3*-HBA (100 mg/kg/d) was administered for 1 week via i.p. injection, and heparin (200 IU) was administered once via i.p. injection. Gross changes in the tail vein 72 h after κ-carrageenan injection. The black bar indicates the position 13 cm from the tail tip. The dot graph represents the change in gross length. Results are expressed as the mean ± standard error. * and ** indicate *p* < 0.05 and *p* < 0.005 compared to the control group, respectively.

To evaluate the vasculoprotective effect of *3*-HBA on rat blood aggregation, *ex vivo* and *in vivo* experiments were performed. For the *ex vivo* experiment, sample blood was collected from SD rat whole blood and mixed with *3*-HBA before aggregation. *3*-HBA decreased aggregation velocity in ADP (20 μM)-induced platelet aggregation in a dose-dependent manner (Fig 5A). For the *in vivo* experiment, *3*-HBA was introduced to rats for 1 week, and the results show that *3*-HBA has the potential to act as an anti-coagulant, similar to aspirin (Fig 5B).

To evaluate the anti-thrombic effect of *3*-HBA, Bekemeier’s modified tail vein thrombosis assays were performed. Three different SD rat groups were injected with substances for 1 week, including *3*-HBA (100 mg/kg, n = 6), heparin (positive control, 200 IU, n = 6), and normal saline (sham-operated, n = 6). For the positive control group, heparin was injected one time along with a 1 mg/kg κ-carrageenan injection. Representative images show the average thrombotic region of each group after 72 h. The black line indicates the tail position 13 cm from the tip. At this position, the tail was tied to induce tail vein thrombosis. The dot plot represents the length of the thrombotic region throughout the different time intervals (Fig 5C).

### *3*-HBA inhibits neointima formation in balloon-injured common carotid arteries (CCAs)

**Fig 6:**
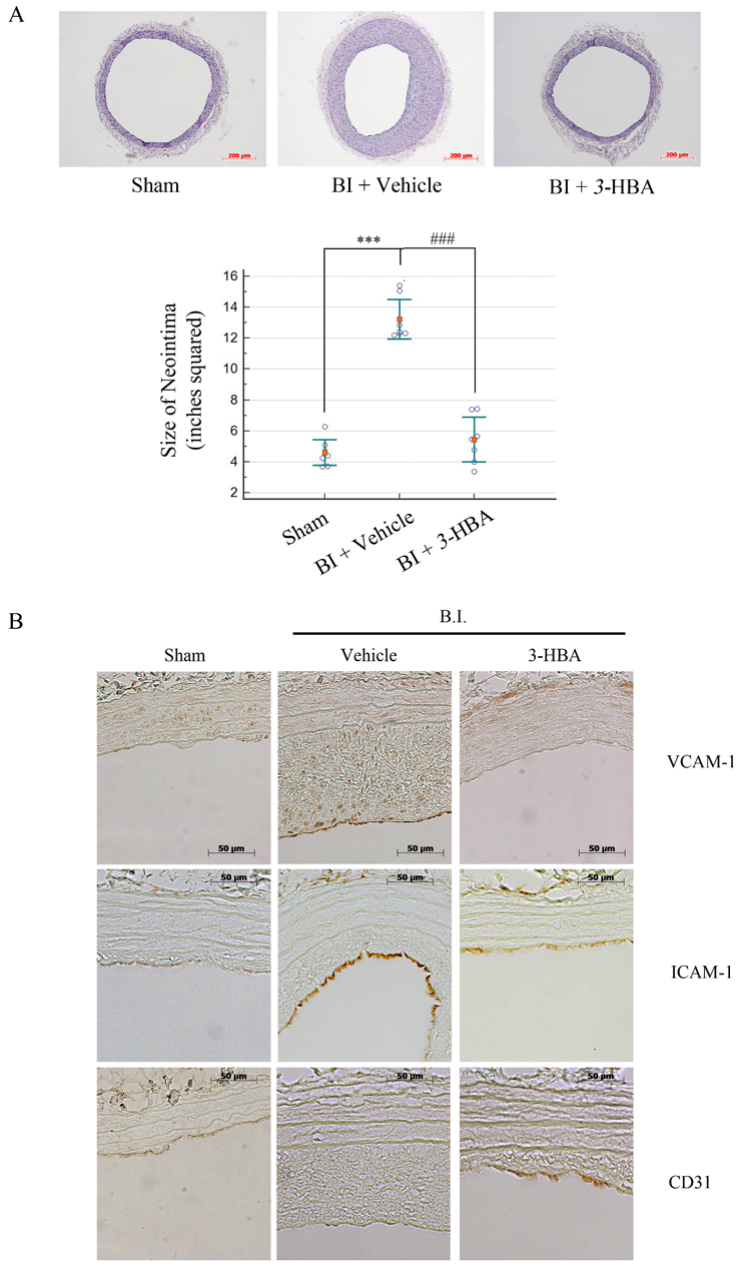
The effect of *3*-HBA in CCA balloon-injured Sprague Dawley rats. (**A, B**) Seven-week-old SD rats (200 g) were treated with the appropriate substance by i.p. injection for 2 weeks, and then common carotid arteries (CCAs) from the rats were balloon-injured. The chosen substance was injected for another 4 weeks, and then the rats were sacrificed for (**A**) hematoxylin and eosin staining of CCAs. The graph shows the percentage of neointima area in SD rats from each group (*n* = 7). Measurements were performed using Scion Image software. (**B**) Immunohistochemistry staining of rat aortas show CD31, VCAM-1, and ICAM-1 production in the linings of the aorta from the same tissues used in Fig 6A. Data are presented as the mean ± SEM. Values represent the mean ± SEM of three experiments; *** indicates *p* < 0.001 compared to the sham group. ### indicates *p* < 0.001 compared to the vehicle group.

The surgical procedure for balloon injury was followed as it is previously described.[12] *3*-HBA was tested for its anti-atherogenic effect against neointima formation. Treatment with *3*-HBA effectively inhibited the formation of neointima compared to vehicle-treated aortas (Fig 6A). Consistent with this observation, neointima size was greatly inhibited in *3*-HBA-treated rats compared to vehicle-treated rats (Fig 6A). Each neointima size was measured by calculating media to lumen ratio of rat aorta. Results show that BI+*3*-HBA aorta has greatly decreased media to lumen ratio compared to BI+Vehicle. The CCAs of rats were immunohistochemically stained with anti-VCAM-1, anti-ICAM-1, and anti-CD31 antibody to determine whether they corresponded with the *in vitro* data obtained from HUVECs (Fig 3B and 3C). VCAM-1 and ICAM-1 staining showed that *3*-HBA treatment dramatically inhibited the inflammation. CD31 staining shows that BI+vehicle group does not have endothelial cells compared to sham group, because of endothelial denudation by balloon injury. However, the treatment of 3-HBA shows partial protection and survival of endothelial cells from balloon injury (Fig 6B).

## Discussion

For the development of therapeutic drugs, compound toxicity must be considered. Toxicity can cause diarrhea, skin irritation, and respiratory issues in addition to other symptoms that were not observed under our *3*-HBA concentration (100 mg/kg/day) conditions. Several findings suggest that the *3*-HBA concentration used in this study is well below toxic conditions. Kluwe et al.[32] have shown that the no-observed-toxic effect dose of benzaldehyde is 300 mg/kg/day in rats and mice, which is three times higher than the concentration used in the present study. Anderson et al.[10] have reported that the intraperitoneal LD(50) for *3*-HBA in rats is 3,265 mg/kg, and the no-observed-adverse-effect level (NOAEL) is 400 mg/kg.

Recent evidence suggests that vascular endothelial inflammatory processes are critical in the initiation of atherosclerosis.[33] Thus, it was necessary to assess the vasoprotective effect of *3*-HBA in endothelial cells. Our findings suggest that *3*-HBA dramatically inhibits the inflammatory responses provoked by TNFα treatment through inhibition of VCAM-1, ICAM-1, p-NF-κB, and p-p38. Inflammation experiments in VSMCs showed additional therapeutic advantages of *3*-HBA in cell migration. No inhibitory effects were observed in ROS assays of VSMCs (data not shown). However, *3*-HBA showed inhibitory effects on MMP-2 (Fig 2B), which is known to be involved in neovascularization, repair of damaged cells, and inflammation in VSMCs,[34, 35] Further researches are needed to verify the downstream molecules influenced by *3*-HBA in VSMCs.

As described earlier, angiogenesis and inflammation are closely associated, and pathologic angiogenesis has been linked with the development of chronic inflammatory diseases. [9] The interaction between angiogenesis and inflammation allows a greater opportunity for leukocyte infiltration to inflammatory sites. Subsequently, this leukocyte infiltration leads to atherosclerosis.[9, 36] The relationship between pathologic angiogenesis and inflammation is best understood through increased vascular permeability, which is observable in chronic inflammation, diabetic retinopathy, solid tumors, myocardial infraction, and wounds.[37] Angiogenic factors such as PDGF and VEGF increase the vascular permeability of microvessels to circulating macromolecules, creating greater probability of inflammation.[9] Our findings on the anti-migrative and anti-angiogenic activity of *3*-HBA in cell migration assay and sprout ring assays, but further researches are needed to confirm the inhibitory effect of *3*-HBA on PDGF signaling and TNFα-induced angiogenesis.

Flavonoids are well-known compounds that readily exhibit antioxidant activity and are easily synthesized from benzaldehyde.[38, 39] A recent report showed that the three ring-structured flavonoids differ in antioxidant activity depending on the number of OH groups. It is reported that OH groups of flavonoids an important role in electron resonance and in electron donation to the oxidizing agent.[21] Other studies have reported that the 3,4–ortho-dihydroxyl group is an important structural requirement for antioxidant activity. These reports suggest the importance of OH group of *3*-HBA in exhibiting vasculoprotective effects. Also, some reports have shown the substitution of OH group by methylation or glycosylation leads to the loss of antioxidant and antiradical activities in flavonoids. This fact is an important clue emphasizing that the presence of the C3-OH is critical in the therapeutic effects of *3*-HBA. Our data also support this conclusion, showing that the C4-OH of *4*-HBA has no therapeutic effects (S3 Fig). Another interesting report showed that the different OH group positions alter oxygen reactive absorbance capacity values. Other reports showed that flavonoids containing 3,4-ortho-dihydroxyl or 3-hydroxyl groups have stronger intracellular antioxidant activity than flavonoids that do not have or have fewer OH groups.[21, 40] This fact corresponds with our previous and current data, in which *3*,*4*-*di*HBA and both *3*-HBA showed antioxidant activity.

The interaction between *3*-HBA and G protein-coupled estrogen receptor-1 (GPER-1) could be possible as it was previously reported from previous reports on protocatechuic aldehyde (PCA) and GPER-1. GPER-1 is an important receptor that has been reported to have involvement in cardiovascular diseases, especially atherosclerosis. Notably, there is structural similarity between PCA and *3*-HBA, differing by only one OH group at the *para* position. Further studies are needed to determine the relationship between GPER-1 and *3*-HBA.

In the present study, we demonstrated for the first time that *3*-hydroxylbenzaldehyde (*3*-HBA) 1) prevents PDGF-induced VSMC migration and proliferation, 2) arrests the S and G0/G1 phases of the cell cycle through Rb1 and CD1, 3) inhibits inflammatory markers (VCAM-1, ICAM-1, p-NF-κB, and p-p38) in HUVECs, 4) prevents ADP-induced thrombus generation and increases blood circulation after formation of thrombus, 5) inhibits angiogenesis *ex vivo* and *in vivo*, and 6) attenuates balloon-injured formation of Neointima.

## Supporting Information

S1 Fig The vasoprotective effect of *3*-HBA on rat VSMCs and HUVECs migration. VSMCs were serum starved for 24 h and then pretreated with *3*-HBA (0, 100 μM) for 24 h. VSMCs were stimulated with PDGF 25 ng/ml for 24 h. Before stimulation, wells were scratched, and scored using Image J. HUVECs were pretreated with *3*-HBA (0, 100 μM) for 24 h and cells were scratched. The migration index score was measured by using Image J. ** indicates *p* < 0.005 compared to the PDGF group. Values represent the mean ± SEM of three independent sets of experiments.
(TIF)

S2 Fig HO-1 and NRF-2 expression by 3-HBA treatment in HUVECs. HUVECs were serum starved for 24 h and then pretreated with *3*-HBA (0, 100 μM) for 24 h. HO-1 and NRF2 gene expression was analyzed by RT-PCR. Values represent the mean ± SEM of three independent sets of experiments.
(TIF)

S3 Fig Representative Matrigel plugs were photographed (*n* = 3 in each group). Method for 4-HBA treatment of Matrigel plug assay was followed as it is indicated in the Materials and Methods.
(TIF)
