# Supplementary figures and images for: Vasculoprotective Effects of 3-Hydroxybenzaldehyde against VSMCs Proliferation and ECs Inflammation
Source: PLoS One. 2016 Mar 22;11(3):e0149394. doi: 10.1371/journal.pone.0149394 (PMC4803227; doi:10.1371/journal.pone.0149394)

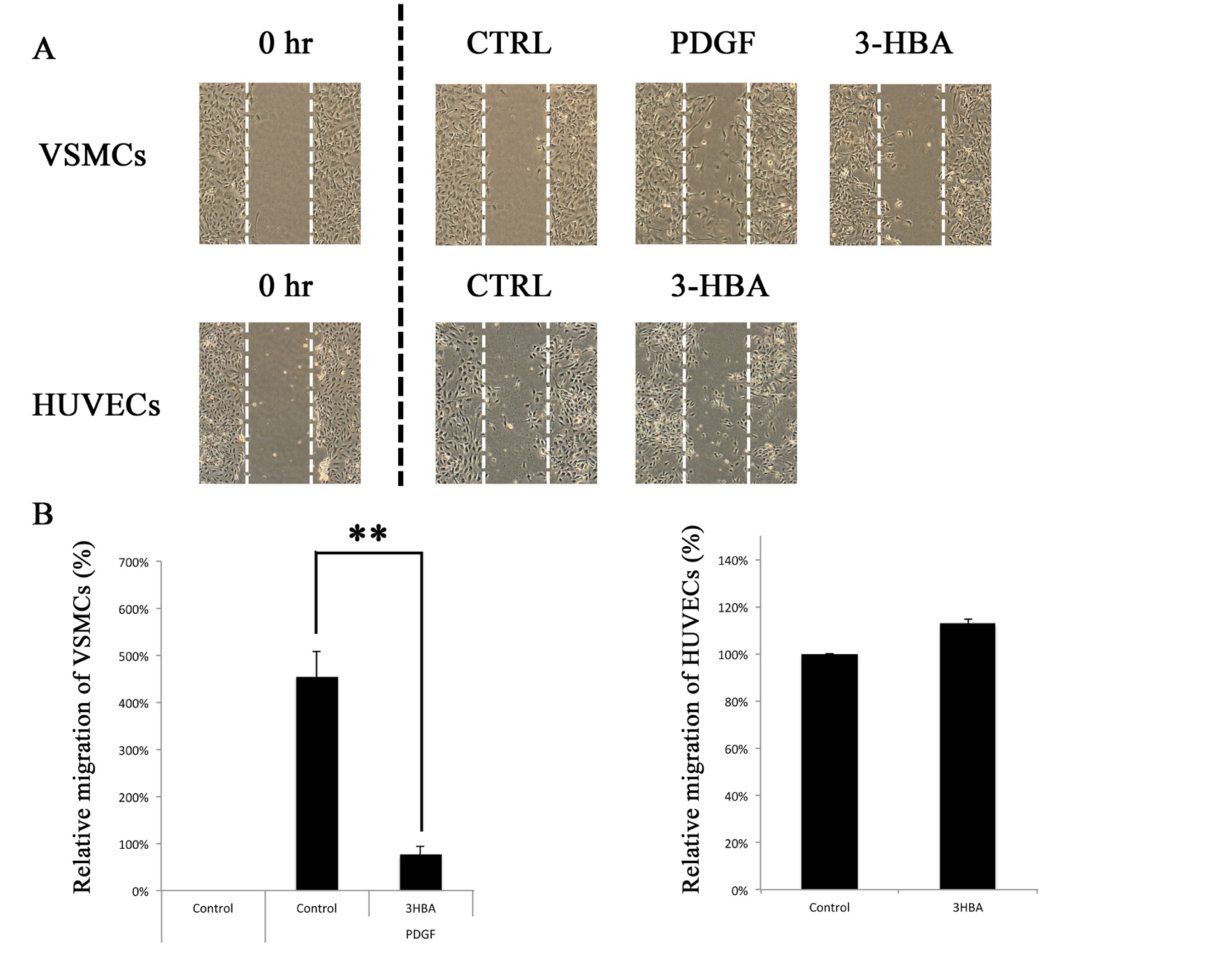

Supplement: S1 Fig — VSMCs were serum starved for 24 h and then pretreated with 3-HBA (0, 100 μM) for 24 h. VSMCs were stimulated with PDGF 25 ng/ml for 24 h. Before stimulation, wells were scratched, and scored using Image J. HUVECs were pretreated with 3-HBA (0, 100 μM) for 24 h and cells were scratched. The migration index score was measured by using Image J. ** indicates p < 0.005 compared to the PDGF group. Values represent the mean ± SEM of three independent sets of experiments. (TIF) [file pone.0149394.s001.tif]

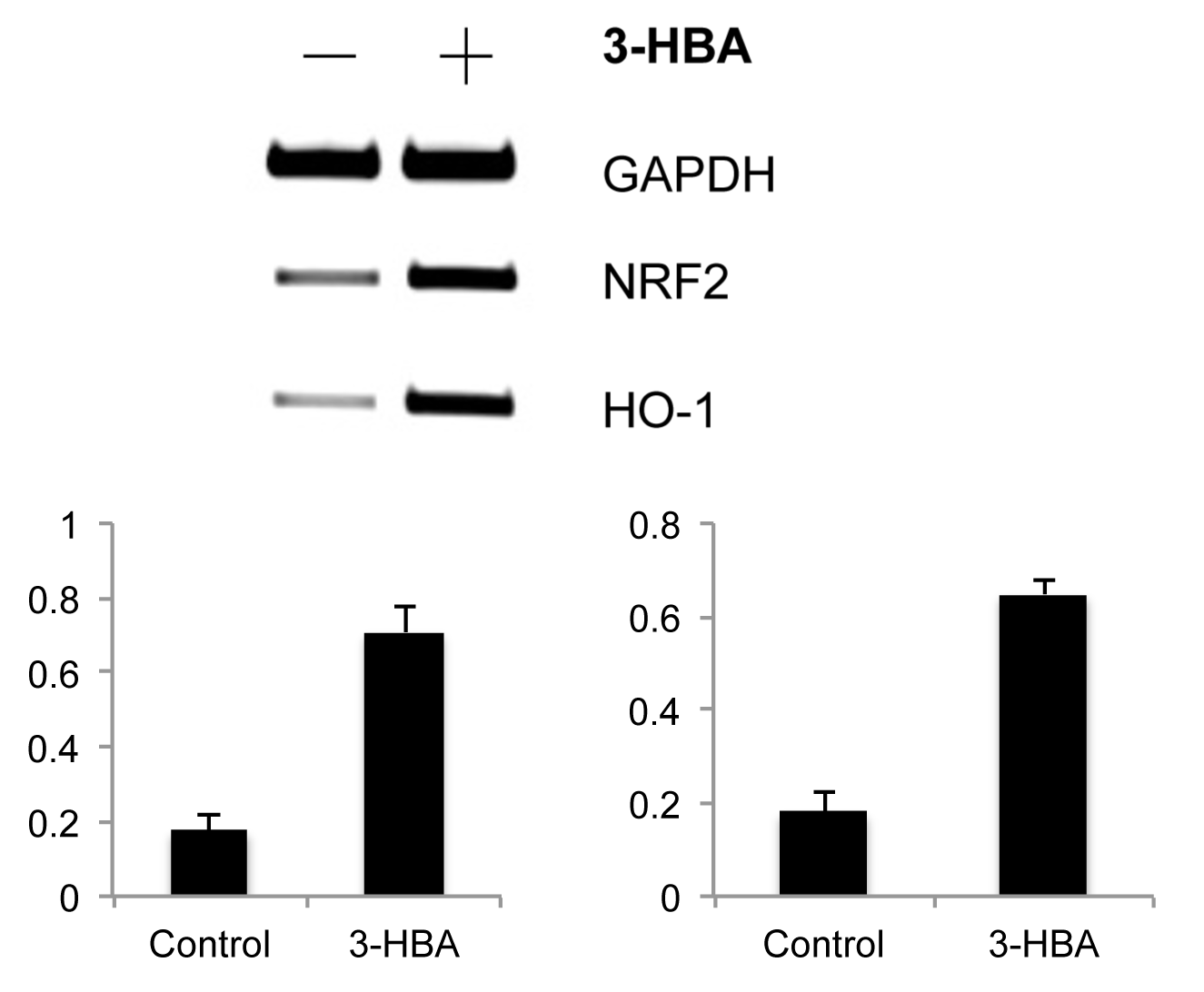

Supplement: S2 Fig — HUVECs were serum starved for 24 h and then pretreated with 3-HBA (0, 100 μM) for 24 h. HO-1 and NRF2 gene expression was analyzed by RT-PCR. Values represent the mean ± SEM of three independent sets of experiments. (TIF) [file pone.0149394.s002.tif]

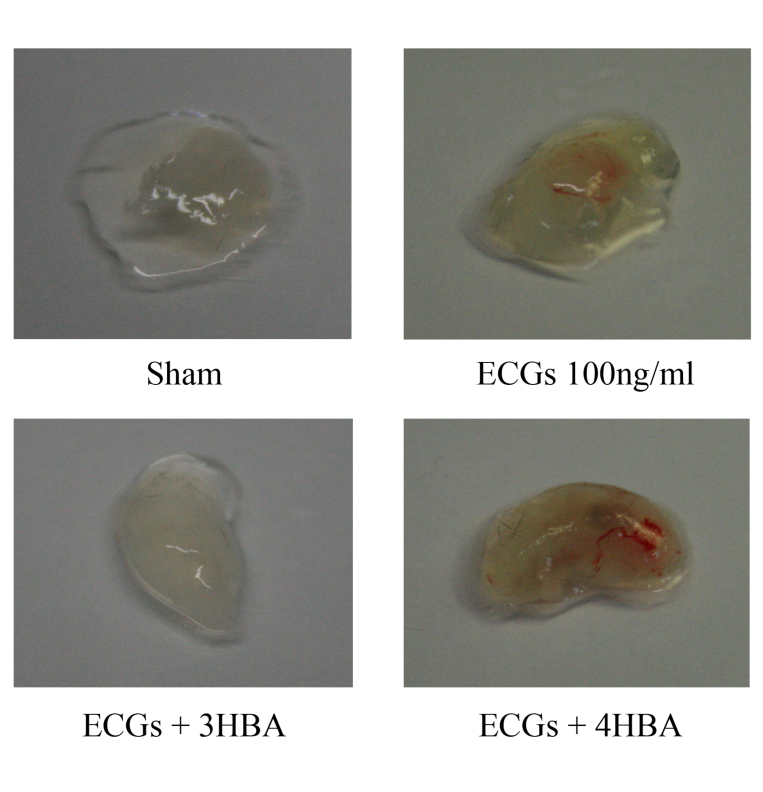

Supplement: S3 Fig — Method for 4-HBA treatment of Matrigel plug assay was followed as it is indicated in the Materials and Methods. (TIF) [file pone.0149394.s003.tif]
